# High carbohydrate diet and physical inactivity associated with central obesity among premenopausal housewives in Sri Lanka

**DOI:** 10.1186/1756-0500-7-564

**Published:** 2014-08-23

**Authors:** Kumari M Rathnayake, Tharrmini Roopasingam, Michael J Dibley

**Affiliations:** Department of Applied Nutrition, Faculty of Livestock, Fisheries and Nutrition, Wayamba University of Sri Lanka, Makandura, 60170 Sri Lanka; Sydney School of Public Health, Sydney Medical School, The University of Sydney, Sydney, Australia

**Keywords:** Central obesity, Premenopausal, Housewives, High carbohydrate diet

## Abstract

**Background:**

Prevalence of obesity and overweight is rising in developing countries, including Sri Lanka at a rapid pace due to dietary and lifestyle changes. This study aimed to assess the association between high carbohydrate diet, physical inactivity and central obesity among premenopausal housewives in Sri Lanka.

**Methods:**

This study was conducted as a cross-sectional study. A sample of 100 premenopausal women with home duties aged between 20 to 45 years were selected randomly from two divisional secretariats (DS), representing urban and rural sectors in Sri Lanka. Data on basic characteristics, anthropometric measurements, dietary assessment and physical activity were collected. We defined central obesity as a waist circumference ≥80 cm, which is the WHO recommended cut-off. Independent sample *t* test was used to compare the mean values. Linear and binary logistic regression analyses were performed to find out the relationship and the magnitude of association between central obesity and percentage of energy contributed from carbohydrate and physical activity level, respectively.

**Results:**

The women reported an average of 18 starch portions per day, which was well above the national recommendations. Seventy percent of energy in the diet came from carbohydrate. The mean BMI and waist circumference of total sample was 25.4 kgm^-2^ and 78.5 cm, respectively. Prevalence of overweight, obesity and centrally obesity among total sample was 38%, 34% and 45%, respectively. A significant positive correlation observed between high carbohydrate diet and waist circumference (r = 0.628, p < 0.0001). There was a significant negative correlation between energy expenditure from physical activity and waist circumference (r = -0.742, p < 0.0001). High carbohydrate diet and physical inactivity were significantly associated with central obesity (OR = 6.26, p = 0.001, 95% CI-2.11-18.57, OR = 3.32, p = 0.028, 95% CI-1.14-9.68).

**Conclusion:**

High carbohydrate diet and physical inactivity are possible explanations for the high prevalence of central obesity. There is an urgent need to implement effective specific public health interventions at household level to reverse this trend among the housewives in Sri Lanka.

## Background

Sri Lanka is a developing country undergoing rapid epidemiological and nutritional transition in which both over-and undernutrition are serious public health concerns. The prevalence of overweight and obesity is increasing rapidly in Sri Lanka. The prevalence of overweight, obesity and central obesity among adults in Sri Lanka was 25%, 9% and 26%, respectively according to the WHO Asian cut-offs [1]. The age-adjusted prevalence of metabolic syndrome among Sri Lankan adults was 24.3% [2]. Moreover, the prevalence of hypertension, diabetes and dysglycaemia in Sri Lanka was 20%, 11% and 20%, respectively [3, 4].

Obesity is a complex mulitfactorial chronic disease [5]. Although obesity increases the risk of chronic non-communicable diseases, studies have shown that the distribution of body fat is more important than general obesity [6]. Moreover, abdominal obesity is an independent predictor of cardiovascular disease risk factors, morbidity and mortality in many populations [7]. In most studies, being a woman is a risk factor for obesity [8]. According to a recent study from Sri Lanka, the prevalence of obesity followed by central obesity was higher in women compared with men [1]. In Sri Lanka, the prevalence of obesity and central obesity based on the WHO cut off values for Asians was 7.2% and 16.5% for men, and 11.5% and 36.3% for women, respectively. The nutritional etiology of obesity remains unclear and controversial especially with regard to the role of dietary and behavioral factors. It is widely accepted that the obesity epidemic in Sri Lanka is partly due to unhealthy dietary habits. The Sri Lankan population mainly depends on cereal-based diets that lack dietary diversity. Based on recent research findings, the average daily serving size of starch consumption among Sri Lankan women exceeded the recommended level of 6–11 servings [9].

Physical inactivity is a well-established risk factor for the development and maintenance of obesity [10]. An association between dietary patterns and behavioral factors and central adiposity has been reported for high-income countries, but data available low and middle-income countries are scanty. Therefore, evaluating the association between the dietary and behavioral factors and central adiposity as a component of metabolic syndrome is of interest.

Since women are more likely to develop obesity and central obesity than men, the present study aimed to assess the association between dietary and behavioral risk factors and central obesity among premenopausal housewives in Sri Lanka.

## Methods

### Subjects and methods

In this cross sectional study a total of 100 premenopausal women aged between 20 to 45 years were selected purposely from two divisional secretariats (DS) namely Negombo and Pannala, representing urban and rural sectors, respectively. The sample population consists of 50 from each DS division including all ethnic groups. Pregnant mothers and lactating mothers were excluded from the study. The study was conducted from January to April 2012.

### Socioeconomic factors

An interviewer-administered questionnaire was used to collect the information about age, monthly family income (low/moderate/high), education level (primary/secondary/tertiary or above) and ethnicity (Sinhalese/Tamils/Muslims).

### Dietary assessment

Dietary intake including all foods and drinks was assessed by a three-day diet diary. It includes two consecutive weekdays and one weekend. Information on their dietary intake was taken in household measurements and was converted to grams by using standard reference tables. Mean total energy intake, percentage of energy from carbohydrate (CHO), protein and fat, and average starch serving size were calculated by using FoodBase 2000 version 2, computerized food composition tables comprising nutrient compositions of Sri Lankan foods.

### Anthropometry

Anthropometric measurements include body weight, height and waist circumference were measured by trained undergraduates using standard equipment according to the standard guidelines [11]. Body weight was measured with a calibrated Seca electronic floor scale accurate to the nearest 0.1 kg while subject was minimally clothed and without shoes. Height was measured to the nearest 0.1 cm with an upright plastic portable Invicta 0955 stadiometer while the subject was in a standing position without shoes. Waist circumference was measured midway between the iliac crest and the lower rib margin at the end of normal expiration using a plastic flexible tape to the nearest 1 cm with the subject having minimum clothing at the waist area. BMI was calculated as weight in kilograms divided by height squared in meters (kgm^-2^). Central adiposity was defined by measuring waist circumference. Definitions for anthropometric cut-offs for overweight, obesity and central obesity were based on WHO cut-offs for Asian women [12].

### Physical activity pattern

Total physical activity pattern for one week was assessed using the short version of the international physical activity questionnaire (IPAQ) [13]. It includes three types of activities vigorous, moderate and time spend for walking. The total amount of energy spent for physical activity was calculated by multiplying the total time spent in each type of activity by the respective MET values, and then adding up these values. The women were categorized into three groups less active/physically inactive (<1500 MET-min/week), moderately active (1500–3000 MET-min/week) and vigorously active (≥3000 MET-min/week) based on the IPAQ cut- off values.

### Statistical analysis

The data were analyzed using SPSS version 16 statistical software package. Descriptive statistical analysis was used to calculate the mean value of the variables. The variables were presented as mean ± standard deviation using 95% confidence intervals. Independent sample *t* test was used to compare the mean values of scale variables among urban and rural sectors. Regression analysis was used to examine the association between central obesity, and percentage of energy contributed from CHO and physical activity level. Multivariate binary logistic regression analysis was performed to assess the magnitude of collective association of risk factors with central obesity. The p value of < 0.05 and the 95% CI range which was not including the value of 1 was considered as significant in logistic regression analysis. High CHO diet was defined as equal to or more than 70% of energy contribution from CHO in the daily diet, which was the mean percent of carbohydrate in the total sample. Moderate and vigorous physically active groups were considered as physically active and less physically active group was considered as physically inactive.

Approval to conduct this study was obtained from the Department of Applied Nutrition, Wayamba University of Sri Lanka, and informed consent was obtained from the subjects before the data was collected.

## Results

Table 1 shows the general characteristics of the study sample. The mean age of the women in the total sample was 33.5 ± 7.4 years. The average family income and monthly expenditure in total sample were LKR 27,580 and LKR 15,625, respectively. They spent nearly 57% of their income for food. Carbohydrate (CHO) was the highest contributor to energy (70%) whereas fat and protein contributed only about 19% and 12%, respectively. The mean energy intake of total sample was 2041 kcal, and the average carbohydrate, protein and fat intakes were about 275, 57and 42 grams, respectively. On average, this study population consumed about 18 portions of starch per day and it was well above the maximum recommendations [14]. Furthermore, there was a significant difference in the percentage of energy contributed from carbohydrate among urban and rural sectors.

**Table 1:** **General characteristics of the study population**

Variables	Urban (n = 50)		Rural (n = 50)		Total (n = 100)		p value
	Mean	± SD	Mean	± SD	Mean	± SD	
Age	32.7	6.5	34.1	8.3	33.5	7.4	0.360
Body weight (kg)	61.3	12.3	60.3	11.9	60.8	12.1	0.668
Body height (cm)	155.4	5.0	153.5	6.8	154.4	6.0	0.116
BMI (kg/m^2^)	25.3	4.5	25.5	4.6	25.40	4.5	0.808
Waist circumference(cm)	80.1	9.9	76.9	9.2	78.51	9.6	0.113
Monthly income (LKR)	29000	1850	23050	1235	27580	1890	0.067
Food expenditure(LKR)	16600	1040	13800	970	15625	1380	0.078
Energy expenditure from physical activity (MET-min/week)	1496.0	694.3	1664.2	772.0	1580.1	735.3	0.255
Energy intake (kcal)	2060.7	343.3	2021.0	374.3	2040.9	357.9	0.581
% of energy from carbohydrate	70.7	4.1	68.2	4.7	69.44	4.5	0.006
% of energy from fat	18.3	3.6	19.1	4.4	18.68	4.1	0.366
% of energy from protein	10.6	1.3	12.7	3.1	11.63	2.6	0.118

P value <0.05 considered as significant in independent sample *t* test.

We identified considerable variation in the frequency of consumption for fruits in the study population. We observed that the overall fruits consumption was not at a satisfactory level. Nearly half of the subjects had consumed fruits only 2–3 times per week whereas one fifth of the sample had consumed fruit only one day per week, and only 20 percent of the women had consumed fruits daily. The prevalence of overweight, obesity and centrally obesity among the study subjects was 38%, 34% and 45%, respectively according to the WHO cut-offs for Asian women [12]. Nearly half of the women were less active and they had very low levels of vigorous physical activity.

**Figure 1:**
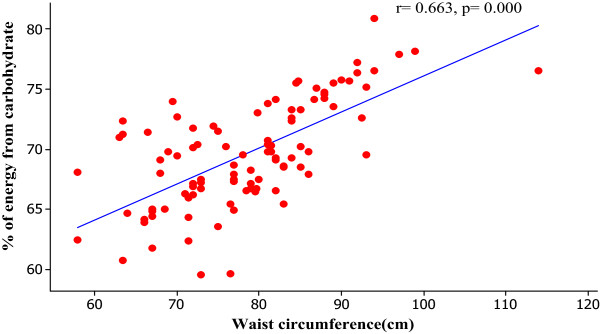
**Association between percentage of energy from carbohydrate and waist circumference.**

**Figure 2:**
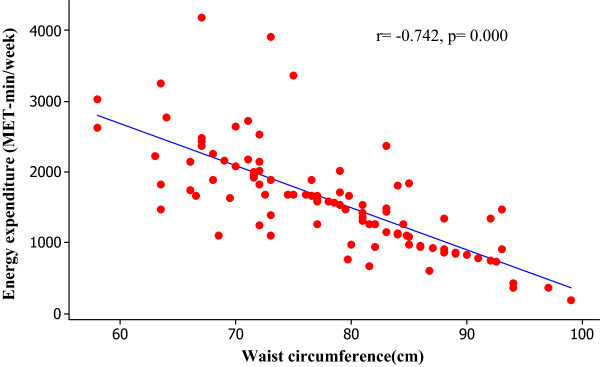
**Association between energy expenditure form physical activity and waist circumference.** (Note: In final analysis of the regression four outliers were excluded).

We assessed the associations between central obesity and the percentage of energy contributed from carbohydrate and energy expenditure from physical activity. We observed positive significant correlation between the percentage of energy contributed from carbohydrate with waist circumference (Figure 1). Moreover, we identified the negative significant correlations between energy expenditure from physical activity with waist circumference (Figure 2). We observed that a high carbohydrate diet (OR 6.26, p value 0.001, 95% CI- 2.11-18.57) and physical inactivity (OR 3.32, p value 0.028, 95% CI- 1.14-9.68) were potential risk factors associated with central obesity among the study population in final multivariate logistic regression analysis. Table 2 shows the odds of being centrally obese with a high carbohydrate diet and physical inactivity.

**Table 2:** **Association between potential risk factors and central obesity (multivariable logistic regression analysis)**

Potential risk factor	Odds ratio	p value	95% CI
Income			
Moderate	1.44	0.665	0.17-3.07
High	8.89	0.011	1.66-30.24
Low (reference)	1.00		
Ethnicity			
Tamil	0.85	0.814	0.22-3.26
Muslim	3.36		0.83-13.58
Sinhala (reference)	1.00		
Frequency of fruit consumption			
Less (<4 days/week)	1.93	0.255	0.62-5.98
High (≥4 days/week) (reference)	1.00		
Percent of energy from carbohydrate			
High (≥70%)	6.26	0.001	2.11-18.57
Low (<70%) (reference)	1.00		
Energy expenditure from physical activity			
Physically inactive	3.32	0.028	1.14-9.68
Physically active (≥1500 MET-min/week) (reference)	1.00		

p value <0.05 and 95% CI value excluding value of 1 were considered as significant.

## Discussion

Sri Lanka is a low-middle income country that is undergoing a rapid epidemiological and nutritional transition. As a result of this rapid socioeconomic transition obesity has become one of the emerging public health concerns among the Sri Lankan adults especially among women. According to a recent national study done in Sri Lanka, the prevalence of obesity, followed by central obesity, was higher in women compared with men [1]. Even though Sri Lanka is a developing country, about the reported mortality from cardiovascular diseases (524 deaths per 100 000) is considerably higher than the rate in developed countries [15]. A recent national study has investigated the risk factors associated with overweight and obesity among Sri Lankan adults in both males and females [1], but little is known about the association between central obesity and the contribution of energy from carbohydrate and physical inactivity among women. Studies suggest that the pattern of body fat distribution is a more important determinant than the general obesity [16]. It is widely accepted that the obesity epidemic in Sri Lanka is partly due to unhealthy dietary habits [15]. Therefore, in this study we investigated the prevalence of obesity and its association with central obesity and the high carbohydrate diet and physical activity to provide baseline information to set up national investigation.

Dietary intake data of the study population indicates nutrition imbalance. We found carbohydrate was the highest contributor to energy intake (70%) while fat only contributed about 19%. The higher percentage of energy from carbohydrates in this study population could be due to the consumption of a rice-based diet for three meals per day. As compatible with a recent study done in Sri Lanka [9] on average, this study population consumed about 18 portions of starch per day, which was well above the maximum recommendations for Sri Lankan adults [14]. Nearly 70% of the study subjects consumed the well above the upper cut-off of the recommendations for the number of daily starch portions. This high starch intake results from the average person’s meal compromising of three-quarters of rice with a small amount of vegetable and some starchy curries. A high-carbohydrate meal leads to negative metabolic imbalances in the body such as hyperinsulinaemia, high serum TAG and low HDL levels [17]. Most Sri Lankans consume the largest carbohydrate portion for lunch or dinner and limit themselves to three meals per day, which may cause postprandial hyperglycaemia and hypertriacylglycerolaemia [17].

Our results showed a low frequency of consumption of fruits among the women participating in the study. The low intake of fruits may be a contributing factor to the high prevalence of obesity in this study population. Studies have shown a reduced risk of obesity with increased fruit and vegetable consumption among women. As fruits and vegetables are rich in dietary fiber and low in glycemic index, they reduce the energy density of the diet and promote satiety [18]. A greater annual increase in waist circumference has been observed in the subjects with white bread dietary pattern that is high in starch, when compared to healthy dietary pattern that is rich in fruits and vegetables [19]. Moreover, a study of Tehranian adults showed a low risk of obesity among women with high dietary diversity scores for fruits and vegetables [20]. In populations from the Middle East dietary patterns including high intake of refined grain white rice and bread in large portion sizes leading to a high contribution of carbohydrate to total food energy are key risk factors for obesity [21]. Comparable to our findings Jayawardena *et al.* also reported the low consumption of fruits among the Sri Lankan adults, who did not consume the number of servings of fruit per day recommended by the national dietary guidelines [14].

The total prevalence of overweight, obesity and central obesity among the women in the study sample was higher than that of a national study conducted in Sri Lanka [1]. But according to the study done by Arambepola *et al*. [22] the prevalence of central obesity in Colombo district in Sri Lanka based on Asian cut-offs was 34.9%. In the present study, we only recruited pre-menopausal middle-aged women and used waist circumference, to assess the central adiposity.

We observed positive significant correlation between percentage of energy contributed from carbohydrate with waist circumference. This suggests an effect of high carbohydrate diet on central adiposity. The important finding of this study is the positive association between high carbohydrate consumption and central obesity. The significantly higher prevalence of central obesity may be due to the high carbohydrate meals they consume. A major concern about high carbohydrate diet is that excess calories will induce production of triglyceride and deposition of them in adipose tissue.

We identified a significant negative correlation between energy expenditure and waist circumference. It indicates the negative association with physical activity and central adiposity. Physical inactivity is another risk factor for development of obesity among Sri Lankan housewives. In this present study, none of them involved in any of the sport activities and therefore more than half of them were categorized as sedentary. This will lead for maintaining a positive energy balance in the body. Studies have reported that housewives involved in repeated housework everyday and this will reduce their energy expenditure in other aerobic physical activities such as walking, jogging, cycling etc. which involve large number of upper body muscles and will lead to fat deposition in abdominal area [6]. Furthermore, housewives have high exposure to food at home and this will lead to increase intake among them. As a result of industrialization the human effort has reduced and dependence in machinery has increased. This leads to reduced energy expenditure and more towards a sedentary life style. The introduction of new technologies in domestic works has reduced the energy expenditure among housewives and makes them sedentary by increasing their television viewing hours [23]. This study supports the importance of public health efforts to strengthen the health messages on physical activity for obese women separately from the promotion of physical activity for weight maintenance (30 minutes/more of moderate to vigorous intense activities on at least 3 days per week). A 45–60 minutes/day of moderate intensity is required to prevent excess weight gain and 60–90 minutes/day moderate activity is essential for weight maintenance of post obese [10]. We did binary logistic regression analysis to find out the possibility of being centrally obese with the high carbohydrate diet and physical inactivity. Women having high carbohydrate diet had more chance of being centrally obese compared with reference group. Indeed, subjects with less physically active had more chance to being centrally obese than the normal subjects.

In a recent national study done by Katulanda *et al.* on prevalence of obesity extensively discussed the risk factors associated with overweight and obesity among in Sri Lankan adults [1]. However, in Sri Lanka the association between dietary factors and physical activity and central obesity is still not revealed specially the dietary and physical activity factors on central obesity. One of the limitations of this study is, we did this study with a sample of premenopausal women representing all ethnic groups and education levels but not from a nationally representative sample. Therefore, future prospective studies are required to confirm these findings. Indeed, the cross sectional design of our study restricts examining causal associations. However, this present study could be considered as the first pilot study for finding association between central obesity and its risk factors among premenopausal housewives.

This study has important implications for health policy in Sri Lanka. Though studies have reported the high consumption of starch portions by Sri Lankan adults, there has been no investigation of the association between a high carbohydrate diet and central obesity. Dietary advice from healthcare professionals offered in clinical and community settings may have over emphasized reductions in fat intake to prevent obesity, and not focused on the effects of a high carbohydrate diet. This present study highlights the high prevalence of obesity in premenopausal unemployed women in Sri Lanka and hence the need for early public health interventions to prevent further worsening of the situation due to the ongoing socioeconomic and nutrition transition of the country.

## Conclusion

In this study, we have shown a high prevalence of central obesity among premenopausal women with home duties in Sri Lanka. High carbohydrate diet and physical inactivity are associated with the occurrence of central obesity. There is an urgent need to implement effective public health interventions to reverse this trend among the premenopausal housewives in Sri Lanka.

## Abbreviations

BMI: Body mass index
CHO: Carbohydrate
DS: Divisional secretariat
IPAQ: International physical activity questionnaire
WHO: World health organization.

## References

[1] Katulanda P, Jayawardena MA, Sheriff MH, Constantine GR, Matthews DR (2010). Prevalence of overweight and obesity in Sri Lankan adults. Obes Rev.

[2] Katulanda P, Ranasinghe P, Jayawardena R, Sheriff R, Matthews D (2012). Metabolic syndrome among Sri Lankan adults: prevalence, patterns and correlates. Diabetol Metab Syndr.

[3] Wijewardene K, Mohideen M, Mendis S, Fernando D, Kulathilaka T, Weerasekara D, Uluwitta P (2005). Prevalence of hypertension, diabetes and obesity: baseline findings of a population based survey in four provinces in Sri Lanka. Ceylon Med J.

[4] Katulanda P, Constantine GR, Mahesh JG, Sheriff R, Seneviratne RDA, Wijeratne S, Wijesuriya M, McCarthy MI, Adler AI, Matthews DR (2008). Prevalence and projections of diabetes and pre-diabetes in adults in Sri Lanka—Sri Lanka diabetes, cardiovascular study (SLDCS). Diabet Med.

[5] Rippe JM, Crossley S, Ringer R (1998). The obesity epidemic: a mandate for a multidisciplinary approach. J Am Diet Assoc.

[6] Folsom AR, Kaye SA, Sellers TA, Hong CP, Cerhan JR, Potter JD, Prineas RJ (1993). Body fat distribution and 5-year risk of death in older women. JAMA.

[7] Prineas RJ, Folsom AR, Kaye SA (1993). Central adiposity and increased risk of coronary artery disease mortality in older women. Ann Epidemiol.

[8] Erosy C, Imamoglu S (2006). Comparison obesity risk and related factors in employed and unemployed (housewife) premenopausal urban women. Diabetes Res Clin Pract.

[9] Jayawardena R, Byrne NM, Soares MJ, Katulanda P, Hills AP (2012). Food consumption of Sri Lankan adults: an appraisal of serving characteristics. Public Health Nutr.

[10] Stamatakis E, Hirani V, Rennie K (2009). Moderate-to-vigorous physical activity and sedentary behaviours in relation to body mass index-defined and waist circumference-defied obesity. Br J Nutr.

[11] World Health Organization Measuring Obesity (1989). Classification and Description of Anthropometric Data.

[12] World Health Organization Expert Consultation (2004). Appropriate Body Mass Index (BMI) for Asian and it population for policy and intervention strategies. Lancet.

[13] Craig CL, Marshall AL, Sjostrom M, Bauman AE, Booth ML, Ainsworth BE, Pratt M, Ekelund U, Yngve A, Sallis JF, Oja P (2003). International physical activity questionnaire: 12-country reliability and validity. Med Sci Sports Exerc.

[14] Nutrition Division, Ministry of Health (2011). Food based dietary guidelines for Sri Lanka.

[15] Abeywardena M (2003). Dietary fats, carbohydrates and vascular disease: Sri Lankan perspectives. Atherosclerosis.

[16] Wei M, Gaskill SP, Haffner SM, Stern MP (1997). Waist circumference as the best predictor of non-insulin-dependent diabetes mellitus compared to BMI, WHR other anthropometric measurements in Mexican Americans: a 7-year prospective study. Obes Res.

[17] Misra A, Khurana L, Isharwal S, Hardwar S (2009). South Asian diets and insulin resistance. Br J Nutr.

[18] Dastgri S, Mahdavi R, Tutunchi H, Faramarzi E (2006). Prevalence of obesity, food choices and socio-economic status: a cross-sectional study in north-west of Iran. Public Health Nutr.

[19] Newby PK, Muller D, Hallfrisch J, Qiao N, Andres R, Tucker KL (2003). Dietary patterns and changes in body mass index and waist circumference in adults. Am J Clin Nutr.

[20] Azadbakht L, Mirmiran P, Esmaillzadeh A, Azizi F (2005). Dietary diversity score and cardiovascular risk factors in Tehranian adults. Public Health Nutr.

[21] Azadbakht L, Mirmiran P, Azizi F (2005). Dietary diversity score is associated with the metabolic syndrome in Tehranian adults. Int J Obes.

[22] Arambepola C, Ekanayake R, Fernando D (2007). Gender differentials of abdominal obesity among the adults in the district of Colombo, Sri Lanka. Prev Med.

[23] Salmon J, Bauman A, Crawford D, Timperio A, Owen N (2000). The association between television viewing and overweight among Australian adults participating in varying levels of leisure-time physical activity. Int J Obes Relat Metab Disord.

